# “The System is Broken”: Caregiver Perspectives of Barriers to Aging in Place with Dementia using the Social Ecological Model

**DOI:** 10.1002/alz70858_098110

**Published:** 2025-12-24

**Authors:** Jessica Russell, Paula C. Fletcher

**Affiliations:** ^1^ Wilfrid Laurier University, Waterloo, ON, Canada

## Abstract

**Background:**

The majority of persons living with dementia in Canada reside at home, relying on support from family and/or friends that act as caregivers. This research examined the lived experiences of persons with dementia and their caregivers while aging in place, using the social ecological model to provide an understanding of various barriers for this group.

**Method:**

Fourteen caregivers were recruited to participate in in‐depth one‐on‐one semi‐structured interviews. Phenomenology was the theoretical orientation used to guide this qualitative research to present an accurate depiction of lived experience. Field notes, member checks, and triangulation were used to enhance the credibility of the study.

**Result:**

The social ecological model provided a framework to classify barriers to aging in place cited by caregivers. The subsequent theme *They don’t make it easy* emphasized issues cited by participants regarding community and societal domains of aging in place care.

**Conclusion:**

Interviews provided knowledge concerning unmet needs and systematic issues faced by caregivers and persons living with dementia and their subsequent impact on care provision. Their stories provide a unique perspective of aging in place with dementia and shed light on the need for reform in health care policies to address systemic barriers and sincerely promote aging in place for *all* persons.

